# Vital Pulp Therapy in Permanent Mature Posterior Teeth with Symptomatic Irreversible Pulpitis: A Systematic Review of Treatment Outcomes

**DOI:** 10.3390/medicina57060573

**Published:** 2021-06-03

**Authors:** João Miguel Santos, Joana F. Pereira, Andréa Marques, Diana B. Sequeira, Shimon Friedman

**Affiliations:** 1 Institute of Endodontics, Faculty of Medicine, University of Coimbra, 3000-075 Coimbra, Portugal; joaninhafpereira@gmail.com (J.F.P.); disequeira@gmail.com (D.B.S.); 2 Health Sciences Research Unit: Nursing, UICISA-E, 3000-075 Coimbra, Portugal; andreamarques23@esenfc.pt; 3 MSc Endodontics Program, Faculty of Dentistry, University of Toronto, Toronto, ON M5G 1G6, Canada; s.friedman@utoronto.ca

**Keywords:** irreversible pulpitis, pulpotomy, calcium silicate cements, vital pulp therapy, systematic review, mineral trioxide aggregate

## Abstract

*Background and Objectives*: Symptomatic irreversible pulpitis in permanent mature teeth is a common indication for nonsurgical root canal treatment (NSRCT), but contemporary studies have reported on vital pulp therapy (VPT) applied in such teeth as a less invasive treatment. This systematic review assessed the outcomes of VPT, including partial and full pulpotomy performed with hydraulic calcium silicate cements (HCSCs) in permanent mature posterior teeth diagnosed with symptomatic irreversible pulpitis. *Materials and Methods*: The PRISMA guidelines were followed. The search strategy included PubMed^®^, EMBASE, Cochrane library and grey literature electronic databases. The quality assessment of the identified studies followed the Cochrane Collaboration Risk of Bias, ROBINS-I and Newcastle–Ottawa Scale tools. *Results*: The search of primary databases identified 142 articles, of which 9 randomized controlled trials and 3 prospective cohort studies were selected for review. The risk-of-bias was assessed as ‘high’ or ‘serious’, ‘fair’, and ‘low’ for three, seven and two articles, respectively. One to five years after VPT using HCSCs, the success rates mostly ranged from 78 to 90%. Based on two articles, the outcomes of the VPT and NSRCT were comparable at one and five years. Despite the necessity for the intra-operative pulp assessment in VPT procedures, the majority of the studies did not fully report on this step or on the time needed to achieve hemostasis. Small sample sizes, of under 23 teeth, were reported in three studies. *Conclusions*: The reviewed 12 articles reported favorable outcomes of the VPT performed with HCSCs in permanent mature posterior teeth with symptomatic irreversible pulpitis, with radiographic success in the range of 81 to 90%. Two articles suggested comparable outcomes of the VPT and root canal treatment. Universal case selection and outcome criteria needs to be established for VPT when considered as an alternative to NSRCT. This evidence supports the need for further research comparing longer-term outcomes of both of the treatment modalities.

## 1. Introduction

Pulpitis is inflammation of the pulp tissue of teeth in response to injurious stimuli, including bacterial invasion of the tooth structure. It typically progresses from a stage considered reversible if the stimulus is removed, to stages considered irreversible. The clinical diagnosis of a healthy pulp, reversible or irreversible pulpitis is based on past and present clinical signs and symptoms, the extent of adjacent caries, responses to pulp testing and percussion, and the radiographic presentation of the periapical tissues at the root apex [1]. Traditionally, pre-operative spontaneous or elicited severe pain and carious pulp exposure are widely considered as indicators of irreversible pulpitis, even though inflammatory changes in teeth exhibiting these symptoms are frequently limited to the coronal pulp next to carious lesions [2,3,4,5,6,7,8], while the majority of the pulp tissue remains uninflamed and viable [9]. Consequently, the traditional classification of irreversible pulpitis has been challenged by the emerging understanding that the removal of the extensively inflamed pulp tissue can allow the remaining uninflamed pulp to be preserved, rendering the pulpitis reversible [1,9]. This emerging understanding has implications on the approaches to the treatment of teeth diagnosed with irreversible pulpitis [1,10].

The traditional treatment modality for permanent mature teeth diagnosed with irreversible pulpitis is pulpectomy, also known as nonsurgical root canal treatment (NSRCT). The major procedural steps and process goals of NSRCT are well established [11,12], including access cavity preparation into the pulp chamber of the tooth, extirpation of the pulp tissue, complete filling of the pulp space to establish an environment conducive to healing and to prevent future bacterial ingress [13], and definitive restoration of the endodontic cavity and coronal tooth structure. While universally accepted, this rather elaborate treatment procedure, often requiring more than one treatment session and a varied armamentarium, may be unattainable [14] when patients cannot afford NSRCT, or when they have no access to skilled treatment providers who can perform it. In those situations, the teeth diagnosed with irreversible pulpitis may be extracted, jeopardizing function and quality of life [15]. When NSRCT meeting the established process goals is performed in teeth diagnosed with irreversible pulpitis, 92 to 98% of the teeth are expected to remain healthy, based on radiographic and clinical criteria, several years after treatment [16,17,18]. In contrast, cross-sectional studies of root-filled teeth in general populations from many countries [19,20,21,22] have reported high prevalence values of inadequate root fillings (56 to 86%) and apical periodontitis (34 to 68%), suggesting that often neither the accepted process goals nor the expected outcomes are achieved.

An alternative to NSRCT for teeth with pulp inflammation is vital pulp therapy (VPT), primarily encompassing direct pulp capping (DPC), partial pulpotomy (PP) and full pulpotomy (FP) procedures [23]. VPT is consistent with contemporary concepts of minimally invasive dentistry [24], and has long been considered a definitive treatment for deciduous teeth with pulp inflammation [12,25] and for permanent immature teeth diagnosed as having reversible or irreversible pulpitis [26,27]. In the past two decades, VPT has been buttressed by the introduction of hydraulic calcium silicate cements (HCSCs), such as mineral trioxide aggregate (MTA), calcium-enriched mixture cement (CEM) and Biodentine™ (Septodont, Saint-Maur-des-Fossés, France), which effectively seal the pulp wound interface and enable pulp healing [28,29,30]. Supported by the current understanding of the capacity of the inflamed pulp tissue to recover and regenerate [1,9], FP and PP using HCSCs have been applied in permanent mature teeth to treat deep carious lesions with exposed pulps, yielding good outcomes [26,31,32,33]. The application of FP has also been extended to permanent mature teeth with symptomatic irreversible pulpitis [2,3,4,5,6,34,35,36,37,38], suggesting that also those teeth, traditionally considered beyond pulpal repair, can be predictably treated by the removal of the inflamed and affected tissue, resulting in maintained vitality of the remaining pulp and periapical health [2,3,4,5,6,7,8,34,35,36,37,38,39,40,41].

The primary aim of this systematic review was to assess the current evidence on treatment outcomes of VPT using HCSCs, applied in permanent mature posterior teeth diagnosed with symptomatic irreversible pulpitis. The study addressed the following research question: “In permanent mature posterior teeth diagnosed with symptomatic irreversible pulpitis, what is the clinical, radiographic or overall outcome after treatment with VPT?” A secondary aim of the study was a comparison of the outcomes between VPT and NSRCT.

## 2. Materials and Methods

PRISMA guidelines [42] were followed. Two reviewers (J.M.S., J.F.P.) established the protocol with input from additional experts (A.M., D.S., S.F.). The principal search was performed in PubMed^®^, EMBASE and Cochrane library electronic databases. It was limited to publications from 2000 to 2021 (PubMed/Medline: 1 January 2000 to 1 March 2021; Embase: 2000–2021; and Cochrane 2000–2021). Free terms and medical descriptors (e.g., MeSH terms) were used for each of “pulpitis”, “irreversible”, “pulpotomy” and “vital pulp therapy”, connected by a Boolean operator “AND” (e.g., PubMed search strategy: “Irreversible” AND “pulpitis” AND “Pulpotomy”[Mesh] OR “pulpotomy”). The principal search was supplemented by grey literature search (Open Grey and ClinicalTrials.gov), cross search in relevant journals for this subject and by references cited in the identified articles and systematic reviews. Specific inclusion criteria (Table 1) were used to select studies for review.

### 2.1. Data Extraction and Analysis

Articles were screened for inclusion in three phases using EndNote^®^ software (Clarivate Analytics, Philadelphia, PA, USA), as follows: (1) Initial search and deletion of duplicates. (2) Two reviewers (J.M.S., J.F.P.) independently screened the electronic search results by title. When a title appeared relevant the abstract was reviewed for eligibility. (3) When the title and abstract were considered relevant the full text of the article was reviewed. A specific data extraction form was created and pilot tested on two studies for feasibility and comprehensiveness, resulting in minor adjustments. Data were then extracted independently by the two reviewers. Disputed selections of articles by the two reviewers were jointly discussed. A third reviewer (A.M.) stood by to arbitrate any remaining disputes.

### 2.2. Recall Rate and Treatment Outcomes

The recall rate was calculated by dividing the number of teeth captured in follow-up examinations by the total number of treated teeth. The primary outcome of interest was ‘success’, defined as absence of clinical signs (sinus tract, swelling, edema or redness) and symptoms (pain, tenderness to percussion or palpation) combined with normal radiographic appearance of the periapical tissues. The success rate for each study was determined by the number of teeth recorded as ‘success’ divided by the total number of treated teeth.

### 2.3. Quality Assessment

Two Cochrane Collaboration Risk of Bias assessment tools were used. The RoB 2 tool was applied to randomized controlled trials and the ROBINS-I tool was applied to non-randomized studies (https://www.riskofbias.info, accessed on 1 September 2020). The Newcastle–Ottawa Scale was additionally applied to cohort studies. The assessments were performed independently by the two reviewers (J.M.S, J.F.P.).

## 3. Results

### 3.1. Selected Studies

**Figure 1:**
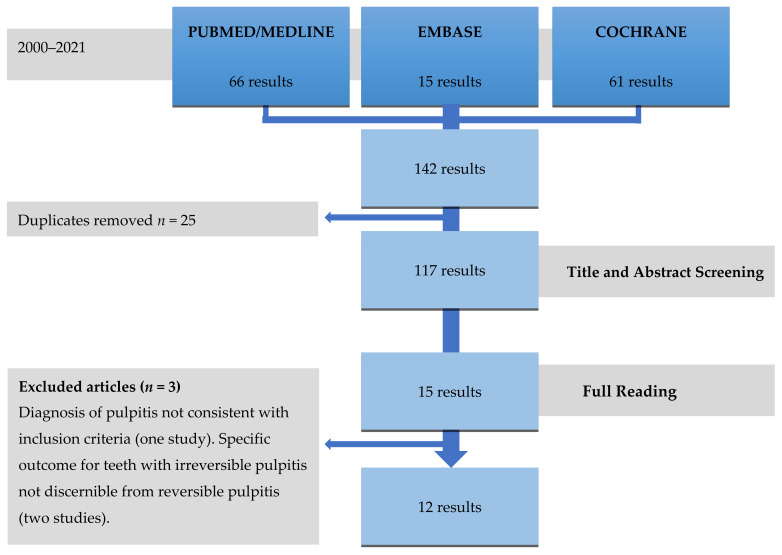
Flowchart of the search strategy and steps of this systematic review.

The main steps of the search and review are outlined in Figure 1. At the conclusion of the electronic search and the exclusion of duplicates, the abstracts of 117 articles were screened, resulting in the exclusion of 102 articles (Supplemental Tables S1–S3). Additional tree articles were excluded after full-text review because the diagnosis of pulpitis was not consistent with the inclusion criteria [43], or the outcome for teeth with irreversible pulpitis was not discernible from the overall sample and attempts to clarify this with the authors were not successful [38,40] (Supplemental Table S4). The hand search and grey literature did not identify any additional articles that met the inclusion criteria. The 12 articles included in this systematic review (Table 2) are characterized in Table 3 and Table 4 according to the study design. Any selection and data extraction disputes were jointly resolved by the two reviewers, with no need for arbitration by the third reviewer.

Eight of the articles [5,6,7,8,34,35,36,37] assessed the outcome of either PP or FP performed with different materials. Three articles [2,3,4] reported on the outcome of FP or NSRCT at three follow-up periods. Two articles [5,6] reported on the outcome of FP performed with different materials at three follow-up periods. Another article reported on the outcome of FP and NSRCT performed in different roots within the same tooth vs. NSRCT performed in all the roots of treated teeth [41]. One subset of subjects treated by FP using CEM appears to have been reported twice [2,5], with a subsequent follow-up of two years [3,6] and five years [4,6]. A query seeking clarification from the principal author on the possible duplication of this group was not answered. In three studies [8,35,36], permanent immature molars were sporadically included in the samples; from those studies, only the data for mature permanent teeth were analyzed in this review. No study reporting on DPC meet the inclusion criteria.

### 3.2. Quality Assessment

Nine reviewed articles followed a randomized controlled trial methodology. Using the Cochrane Collaboration Rob 2 tool (Supplemental Tables S5 and S6), the final risk of bias for this article was assessed as ‘low’ for only one [8], as ‘fair’ for seven [2,3,4,5,6,7,41], and as ‘high’ for one [34]. Of the three reviewed prospective cohort studies assessed with the Cochrane Collaboration ROBINS I tool (Supplemental Table S7), the risk of bias was assessed as ‘low’ for two [36,37] and as ‘serious’ for one [35]. When the same three studies were assessed with the Newcastle–Ottawa Scale (Supplemental Table S8), one study each scored four [35], five [36] and six [37] out of a maximum of nine stars. The results of the two articles assessed as having ‘high’ [34] or ‘serious’ [35] risk of bias were considered secondary to those of the remaining 10 articles.

### 3.3. Outcomes

Articles have reported one-year outcomes [2,5,34,36,37,41], two-year outcomes [3,6,7], and 3-to-5-year outcomes [4,6,8,35] (Table 3 and Table 4). At one-year, high clinical success rates ranging from 92 to 100%, and high radiographic success rates ranging from 92 to 98%, have been reported for FP using either MTA, CEM or Biodentine™ [2,5,36,37]. A lower overall success rate of 83% was reported for PP using ProRoot^®^ MTA white (Dentsply Maillefer, Ballaigues, Switzerland) [7]. An even lower overall success rate of 55% was reported for PP using calcium hydroxide [7], but this procedure was not within the scope of this review. Notably, the 83% clinical and 53% radiographic success rates of FP using ProRoot^®^ MTA reported in one study [34] were substantially lower than those of the other studies, but these data are undermined by the lower than desired recall rate and the high risk of bias in this study.

At two years, the clinical and radiographic success rates reported for FP using CEM were 98% and 86%, respectively [3,6]. The overall success rate of PP using ProRoot^®^ MTA was 85% [7]; it was only 43% for PP using calcium hydroxide, but this procedure was not within the scope of this review.

At 3 to 5 years, clinical success rates of 85% and 90%, radiographic success rates of 92% and 100%, and overall success rates of 85% and 90%, were reported for PP using ProRoot^®^ MTA and Biodentine™, respectively [8]. The clinical success rate of FP using CEM or ProRoot^®^ MTA was 98%, while the radiographic success rates were 78% and 85%, respectively [6]. The overall success rates of FP using CEM and ProRoot^®^ MTA were also reported as 78% [4] and 100% [35], respectively; however, the validity of these data is undermined by a low recall rate in one study [4] and a ‘serious’ risk of bias in the other [35].

The outcomes of FP using CEM and of NSRCT were compared at three follow-up periods [2,3,4]. A high clinical success above 97% was reported for both treatment procedures at one year [2] and two years [3], while the radiographic success rate was lower for NSRCT than for FP. At five years [4], the overall success rates of 78% and 75% did not differ significantly between FP and NSRCT, but the validity of the data is undermined by a low recall rate. The one-year overall success of FP performed with MTA in molar roots with vital pulps, while NSRCT was performed in the other roots, was 93%, compared to 90% for NSRCT performed in both roots of the molars [41].

## 4. Discussion

All the reviewed studies used similar diagnostic criteria for irreversible pulpitis, including prolonged spontaneous or elicited pain. The American Association of Endodontists defines both asymptomatic and symptomatic irreversible pulpitis as “a clinical diagnosis based on subjective and objective findings indicating that the vital inflamed pulp is incapable of healing” [44]. The evidence from the present systematic review refutes this notion and suggests that permanent mature teeth diagnosed with symptomatic irreversible pulpitis can be effectively treated by VPT procedures, including FP and PP, corroborating the conclusions of previous systematic reviews [31,32,33,39].

This systematic review was performed using universally accepted guidelines. The search followed PRISMA guidelines [42] and was limited to contemporary publications from 2000 to 2021. The narrow inclusion criteria restricted this review to permanent mature posterior teeth with the diagnosis of symptomatic irreversible pulpitis treated by FP, PP or a combination of FP and NSRCT, while excluding data on mixed pulpal diagnoses, immature teeth and VPT using other materials. The quality of the selected studies was assessed following two Cochrane risk of bias and Newcastle–Ottawa Scale assessment tools. Unlike in two previous systematic reviews [33,39], no consideration was given to the level of evidence of the reviewed studies, primarily because the small sample sizes of 13 to 23 teeth in three of the studies [8,35,36] obscured the distinction between a case series and cohort study design, but also because this review did not aim to develop treatment recommendations for specific procedures. As well as this, a meta-analysis of the studies’ results was considered inappropriate because of the heterogeneity [45] that would undermine the interpretations of the results.

This study provided an insight into the outcomes of FP and PP in permanent mature posterior teeth with symptomatic irreversible pulpitis at several follow-up periods. The outcomes of both procedures, performed with HCSCs, remained mostly consistent in the period of one year [2,5,36,37], two years [3,6,7] and up to five years [4,6] after treatment, with overall success rates in the range of 78 to 90% [6,7,8]. The validity of the reported lower and higher success rates is undermined by a low recall rate [4] and substantial risk of bias [35] suggested by the assessment with the Cochrane Collaboration ROBINS I tool and the Newcastle–Ottawa Scale. The favorable outcomes of VPT in permanent mature teeth corroborated previous systematic reviews [31,32,33,39]. They also appeared to correlate well with the histologic reports of a reparative dentin bridge formation under the dressing material and subjacent healthy pulp tissue after pulpotomy in teeth with irreversible pulpitis [10,29,46]. The outcomes were comparable to those reported for permanent immature teeth [26,27], suggesting that mature pulps were equally amenable to recovery from inflammatory changes.

The reviewed studies captured two approaches to pulpotomy, namely, FP [2,3,4,5,6,34,35,36,37] and PP [7,8]. While it has been suggested that FP may remove inflamed tissue more predictably [26], the comparable outcomes of FP and PP, with the overall success rate above 81%, suggested that both procedures might be considered predictable when carried out with precision. Although not within the scope of this review, the outcomes of FP and PP performed with calcium hydroxide were captured in two studies [7,34], along with the outcomes of the same procedures performed with HCSCs materials. Calcium hydroxide had a poorer outcome compared with MTA in one PP study [7], corroborating previous reports of poorer VPT outcomes for calcium hydroxide than HCSCs materials [14,28,30]. The validity of equivalent FP outcomes reported for MTA and calcium hydroxide in the other study [34] might be undermined by its ‘high’ risk of bias.

The current evidence comparing VPT and NSRCT is limited. A recent randomized controlled trial reported comparable one-year overall success rates of 93% and 90% for FP performed with MTA in one root while NSRCT was performed in the other root of the same tooth, and for NSRCT performed in both roots, respectively [41]. Another randomized controlled trial assessed the outcomes at three follow-up periods [2,3,4]. While differences in radiographic success were reported after one and two years [2,3], the comparable overall five-year success rates of 78% and 75% for FP and NSRCT, respectively, suggested that both might be considered as alternative treatments for permanent mature teeth with symptomatic irreversible pulpitis [4]. These findings notwithstanding, the reported 75% overall success for NSRCT [4] starkly contrasts with the well-established current best evidence for NSRCT [16,17,18] reporting 92 to 98% success two to six years after the treatment of teeth with no pre-operative apical periodontitis. This considerable discrepancy in outcomes, and a low recall rate, undermine the validity of the VPT–NSRCT comparison study [4].

The consideration of a mature permanent tooth as a candidate for VPT requires careful assessment of the extent of damage to the pulp tissue, using clinical and radiographic measures [47]. The pulp condition, routinely surmised pre-operatively from past and present signs and symptoms and responses to clinical tests, must be confirmed intra-operatively by the assessment of pulpal bleeding, tissue color and consistency [48]; if pulp exposure does not elicit bleeding, the coronal pulp may be infected and necrotic, contraindicating a VPT procedure [10]. The significance of intra-operative pulp assessment is well illustrated in one study [37], where the pulps of 7/64 (11%) of teeth diagnosed with irreversible pulpitis were intra-operatively determined as partially (3 teeth) or fully (4 teeth) necrotic, suggesting that pre-operative assessment alone may overestimate the pulp’s amenability to VPT procedures. Importantly, despite the necessity for intra-operative pulp assessment in VPT procedures, the majority of the reviewed studies [2,3,4,5,6,34,35] have not fully reported on this step being performed. Pre-operative radiographic assessment may also be ambiguous. On the one hand, periapical findings may not be present even when the pulp is necrotic [49]. On the other hand, the presence of periapical findings, traditionally considered a pathognomonic sign of pulp necrosis [50], may accompany inflamed pulps that are indeed amenable to repair, as observed in several of the reviewed studies [2,5,35,37]. Thus, the presence of pre-operative radiographic findings may underestimate the pulp’s amenability to VPT procedures.

The correlation between pulpal bleeding time and outcome of VPT procedures has been elusive. Several of the reviewed articles [2,3,4,5,6,34] have not reported the time used for hemostasis. The remaining six articles suggested times for hemostasis ranging from to 2 to 3 min [7], to 4 [36], 6 [37], 8 [41], 10 [8] or even 25 min [35]. Thus, guidelines suggesting that failure to control bleeding after 2 min should be considered a contraindication to pulpotomy [10] are not supported by the reviewed studies. Local anesthetics and their vasoconstrictor concentrations also vary among the studies, including 2% lidocaine with 1:80,000 epinephrine [2,3,4,5,6,7,35,36], 2% lidocaine with 100,000 epinephrine [41] and 4% articaine with 1:100,000 epinephrine [8,37]. Because the composition of the anesthetic used may impact hemostasis, it may directly influence the intra-operative assessment of the pulp condition. As well as this, the best protocol for hemostasis is yet to be established, with all of saline solution, different concentrations of sodium hypochlorite, hemostatic agents, chlorhexidine, corticosteroid paste, and compression with a dried cotton pellet reportedly used for this purpose [47].

The influence of the patient’s age on the prognosis is also unclear. Several of the reviewed studies included only young patients with ages ranging from 9 to 17 years, [8,35,36] up to 35 years [34,41], reflecting the rationale that pulps of young patients possess a higher capacity for recovery. Other studies [2,4,5,6,37], involving a wider range of ages from 9 to 69 years, did not report an impact of the patient’s age on the outcome of VPT.

The outcome of VPT in the reviewed studies has been assessed using a combination of clinical and radiographic measures, as is the routine procedure. With one exception [8], the clinical success was slightly better than the radiographic success [2,3,5,6,35,36,37]. Clinical success was primarily based on the absence of patient-reported symptoms, with additional consideration of probing depth, integrity of the restoration, discoloration, and mobility in several studies [7,8,36,37,41]. A positive response to the cold test was required in one study [7]. Importantly, symptoms may be absent even when the pulp disease progresses to necrosis [10], while the deep restoration and deposition of tertiary or reparative dentin may undermine the reliability of pulp sensibility tests [45]. As well as this, the response of pulpotomized teeth to pulp sensibility testing is unreliable [24]. Radiographic success commonly reflects the absence of findings in the periapical bone [31,32,39], which are subject to interpretation and not easily detected by periapical radiographs in the early stages of tissue breakdown [51]. Inspecting the tooth for the formation of a dentinal bridge under the pulp capping material [8,48] may not be a reliable outcome measure either because of low mineralization, overlapping roots and restorative materials [45]. Indeed, the formation of a dentinal bridge was not considered for the outcome assessment in any of the reviewed studies. Thus, in a short-term after VPT procedures, both the clinical and radiographic outcome assessment have only limited sensitivity in detecting pulpal and periapical disease processes.

Despite the favorable outcomes of VPT in permanent mature teeth with irreversible pulpitis captured in this review and a previous one [39], universal goals, case selection and outcome criteria need to be established for VPT when considered as an alternative to NSRCT [45]. As well as this, because the outcome of VPT depends on several procedural factors, such as the anesthetics used, removal technique of carious tissue, strict disinfection and bleeding control [48], dressing materials [7], type of permanent restoration [47], and possibly the use of magnification [10], the reports from different geographical areas and clinical settings are required to validate the universal applicability of VPT for permanent mature teeth with symptomatic irreversible pulpitis.

## 5. Conclusions

This systematic review captured 12 articles reporting favorable outcomes of full and partial pulpotomy performed with hydraulic calcium silicate cements in permanent mature posterior teeth diagnosed with symptomatic irreversible pulpitis. Two articles suggested comparable outcomes of full pulpotomy and root canal treatment. The missing data on intra-operative pulp assessment to confirm its status, differences in indications and outcome criteria, and small samples undermined the validity of the findings. While limited, this evidence provided the ethical foundation for further randomized controlled trials or large-scale pragmatic trials designed to compare longer-term outcomes of both alternative treatment modalities. As both modalities differ in their degree of invasiveness, the cost and, possibly, accessibility to care, well-designed future studies are critically needed to strengthen the evidence base that can be used by clinicians to inform patients on their options for treatment of permanent mature teeth with irreversible pulpitis.

## Figures and Tables

**Table 1:** Inclusion and exclusion criteria used to qualify studies for review.

Inclusion Criteria	Exclusion Criteria
Human clinical study; Procedures performed in permanent mature posterior teeth; Diagnosis of symptomatic irreversible pulpitis; Procedures include DPC, PP or FP; Outcome measures based on radiographic and clinical criteria; Follow-up period of 1 year or longer; Abstract available; Randomized controlled trial; Prospective cohort study; Definitive coronal restorations present at follow-up; Publication in English, Spanish, French or Portuguese.	Animal study; Human histologic study; Procedures performed in deciduous, permanent immature, or anterior teeth; Diagnosis of asymptomatic irreversible pulpitis or reversible pulpitis; Only clinical or radiographic outcome reported; Preoperative presence of root resorption or calcifications; Retrospective cohort study; Case series; Previously reported cohorts; Narrative review; Survival study.

DPC—direct pulp capping; PP—partial pulpotomy; FP—full pulpotomy.

**Table 2:** Articles included in this systematic review.

Authors, Year	Title	Design
Asgary et al. 2013 [2]	One-year results of vital pulp therapy in permanent molars with irreversible pulpitis: an ongoing multicenter, randomized, non-inferiority clinical trial	Randomized controlled trial
Asgary et al. 2014 [3]	Two-year results of vital pulp therapy in permanent molars with irreversible pulpitis: an ongoing multicenter, randomized clinical trial	Randomized controlled trial
Asgary et al. 2015 [4]	Five-year results of vital pulp therapy in permanent molars with irreversible pulpitis: a non-inferiority multicenter, randomized clinical trial	Randomized controlled trial
Asgary & Eghbal 2013 [5]	Treatment outcomes of pulpotomy in permanent molars with irreversible pulpitis using biomaterials: a multi-center, randomized controlled trial	Randomized controlled trial
Asgary et al. 2017 [6]	Long-term outcomes of pulpotomy in permanent teeth with irreversible pulpitis: a multicenter, randomized controlled trial	Randomized controlled trial
Kumar et al. 2016 [34]	Comparative evaluation of platelet-rich fibrin, mineral trioxide aggregate, and calcium hydroxide as pulpotomy agents in permanent molars with irreversible pulpitis: A randomized controlled trial	Randomized controlled trial
Qudeimat et al. 2017 [35]	Mineral trioxide aggregate pulpotomy for permanent molars with clinical signs indicative of irreversible pulpitis: a preliminary study	Prospective cohort study
Taha & Abdulkhader 2018 [36]	Full pulpotomy with Biodentine in symptomatic young permanent teeth with carious exposure	Prospective cohort study
Taha & Abdelkhader 2018 [37]	Outcome of full pulpotomy using Biodentine in adult patients with symptoms indicative of irreversible pulpitis	Prospective cohort study
Taha & Khazali 2017 [7]	Partial pulpotomy in mature permanent teeth with clinical signs indicative of irreversible pulpitis: A randomized clinical trial	Randomized controlled trial
Uesrichai et al. 2019 [8]	Partial pulpotomy with two bioactive cements in permanent teeth of 6-to-18-year-old patients with signs and symptoms indicative of irreversible pulpitis: a non-inferiority randomised controlled trial	Randomized controlled trial
Koli et al. 2021 [41]	Combination of nonsurgical endodontic and vital pulp therapy for management of mature permanent mandibular molar teeth with symptomatic irreversible pulpitis and apical periodontitis	Randomized controlled trial

**Table 3:** Characteristics of included randomized controlled trials.

Article	Study Arm	Study Sample (n)		Recall Rate (%)	Pulpitis Diagnosis	Age Range (years)	Time for Bleeding Control	Success Rate (%) at Different Follow-Up Periods		
		Initial	Final					1 year	2 years	>2 years
Asgary et al. 2013 [2] 2 study arms	FP CEM	205	167	84.0%	Spontaneous pain for a few seconds to several hours, pain exacerbating with hot and cold fluids, radiating pain, or reproducible pain with cold testing	9–65	NR	C: 97.6% R: 92.2%	-	-
	NSRCT	202	175					C: 98.3% R: 70.3%	-	-
Asgary et al. 2014 [3] 2 study arms	FP CEM	205	166	81.6%	Spontaneous pain for a few seconds to several hours, pain exacerbating with hot and cold fluids, radiating pain, or reproducible pain with cold testing	9–65	NR	-	C: 98.2% R: 86.1%	-
	NSRCT	202	166					-	C: 98.2% R: 79.5%	-
Asgary et al. 2015 [4] 2 study arms	FP CEM	205	137	66.6%	Spontaneous pain for a few seconds to several hours, pain exacerbating with hot and cold fluids, radiating pain, or reproducible pain with cold testing	9–65	NR	-	-	5 years O: 78.1%
	NSRCT	202	134					-	-	5 years O: 75.3%
Asgary & Eghbal 2013 [5] 2 study arms	FP MTA	208	179	83.7%	Spontaneous pain for a few seconds to several hours with extensive caries; pain exacerbated with hot and cold fluids and/or radiating pain	9–65	NR	C: 98.3% R: 95.0%	-	-
	FP CEM ***	205	167					C: 97.6% R: 92.2%	-	-
Asgary et al. 2017 [6] 2 study arms	FPMTA	208	2 years 178 5 years 154	2 years 83.2% 5 years 73.6%	Spontaneous pain for a few seconds to several hours with extensive caries; pain exacerbated with hot and cold fluids and/or radiating pain	9–65	NR	-	C: 98.9% R: 94.9%	5 years C: 98.1% R: 84.6%
	FP CEM ***	205	2 years 166 5 years 150					-	C: 98.2% R: 86.1%	5 years C: 98.0% R: 78.1%
Kumar et al. 2016 [34] 3 study arms	FP ProRoot MTA	19	15	75.9%	Spontaneous, lingering pain, exacerbated by hot and cold fluids, and/or radiating pain	14–32	NR	C: 83.3% R: 53.3%	-	-
	FP PRF	17	13			14–32		C: 92.8% R: 38.4%	-	-
	FP CH	18	13			14–23		C: 81.2% R: 46.1%	-	-
Taha & Khazali 2017 [7] 2 study arms	PP ProRoot MTA White	27	26	98.1%	Severe spontaneous lingering pain that could be reproduced by cold stimuli	20–52	A pellet moistened with 2.5% NaOCl for 2 min and repeated as needed	O: 83.0%	O: 85.0%	-
	PP CH	23	23					O: 55.0%	O: 43.0%	-
Uesrichai et al. 2019 * [8] 2 study arms	PP ProRoot MTA	13	13	100%	Spontaneous pain together with sharp and lingering pain with cold testing	8–17	Up to 10 min	-	-	35 ± 14 months O: 85.0% C: 85.0% R: 92.0%
	PP Biodentine	10	10							45 ± 18 months O: 90.0% C: 90.0% R: 100%
Koli et al. 2021 ** [41] 2 study arms	FP MTA + NSRCT	30	28	100%	Positive response to a pulp sensibility test with periapical index score ≥ 3	18–35	Up to 8 min	O: 93.3%		
	NSRCT	30	27					O: 90%		

FP—full pulpotomy; PP—partial pulpotomy; NSRCT—non-surgical root canal treatment; C—clinical outcome; R—radiographic outcome; O—overall outcome; NR—not reported; CEM—calcium-enriched mixture; CH—calcium hydroxide; MTA—mineral trioxide aggregate; PRF—plasma-rich fibrin. * The authors provided specific information upon request on mature teeth with symptomatic irreversible pulpitis. ** FP was performed in roots assigned a periapical index score of ≤2 and observed having vital pulp tissue in the canal(s). NSRCT was performed in roots assigned a periapical index score of ≥3. *** This study arm appears similar to that reported elsewhere [2,3,4]. A query seeking clarification from the principal author on possible duplication of this study arm was not answered.

**Table 4:** Characteristics of included prospective cohort studies of full pulpotomy (FP).

Author, Year	Pulpotomy Material	Study Sample (n)		Recall Rate	Pulpitis Diagnosis	Age Range (years)	Time for Bleeding Control	Success Rate (%) at Different Follow-Up Periods		
		Initial	Final					1 year	2 years	>2 years
Qudeimat et al. 2017 * [35]	ProRoot MTA Grey and White	13	13	100% **	Intermittent or spontaneous pain; rapid exposure to dramatic temperature changes elicited heightened and prolonged episodes of pain even after the thermal stimulus has been removed	10–13	5 to 25 min	-	-	Mean 57.2 months O: 100%
Taha & Abdulkhader 2018 * [36]	Biodentine	17	17	100%	Spontaneous pain or pain exacerbated by cold stimuli and lasting for a few seconds to several hours interpreted as lingering pain compared with the control teeth	9–17	4 min	C: 100% R: 94.1%	-	-
Taha & Abdulkhader 2018 [37]	Biodentine	64	60	93.7%	Pain or symptoms classically indicative of symptomatic irreversible pulpitis according to AAE diagnostic terminology	19–69	Up to 6 min	C: 100% R: 98.4%	-	-

C—clinical outcome; R—radiographic outcome; O—overall outcome; * Only specific data on mature teeth are presented; ** A specific end point of the follow-up period is not presented.

## Data Availability

Data will be made available upon request to the corresponding author.

## Supplementary Materials

The following are available online at https://www.mdpi.com/article/10.3390/medicina57060573/s1, Supplementary Table S1: Articles excluded after title and abstract screening using PubMed/Medline Database, Table S2: Articles excluded using the EMBASE database, Table S3: Articles excluded using the Cochrane database, Table S4: Articles excluded after full text reading, Table S5: Risk-of-bias in randomized controlled trials based on the Cochrane Collaboration RoB 2 tool, Table S6: Risk of bias assessment justification for randomized clinical trials, Table S7: Risk-of-bias in prospective cohort studies based on Cochrane Collaboration ROBINS-I tool, Table S8: Risk-of-bias in prospective cohort studies based on the Newcastle-Ottawa Scale.
