# Supplementary material for: Vital Pulp Therapy in Permanent Mature Posterior Teeth with Symptomatic Irreversible Pulpitis: A Systematic Review of Treatment Outcomes
Source: Medicina (Kaunas). 2021 Jun 3;57(6):573. doi: 10.3390/medicina57060573 (PMC8228104; doi:10.3390/medicina57060573)
Supplement: Supplementary file 1 [file medicina-57-00573-s001.zip › medicina-1229937-supplementary.pdf]

Table S1. Articles excluded after title and abstract screening using PubMed/Medline Database.

Articles	Cause of exclusion
Eren *et al.* 2018<br>Kérourédan *et al.* 2017<br>Brignardello-Petersen 2017<br>Bane *et al.* 2016<br>Asgary & Eghbal 2010<br>Nyerere *et al.* 2006	Only clinical outcome available
Asgary & Ramazani 2018<br>Asgary *et al.* 2017<br>Ashraf *et al.* 2017<br>Soni 2016<br>Asgary *et al.* 2016<br>Asgary & Kemal 2015<br>Solomon *et al.* 2015<br>Asgary 2011	Case report
Wang *et al,* 2020<br>Sabbagh *et al.* 2016<br>Harandi *et al.* 2013	Case report<br>Immature permanent teeth
Peng *et al.* 2015	Chinese language<br>Immature permanent teeth
Chen *et al.* 2020<br>Ghaderi *et al.* 2020<br>Memarpour *et al.* 2016<br>Parisay *et al.* 2015<br>Whaterhouse *et al.* 2002	Primary teeth
Mousavi *et al.* 2016<br>Mente *et al.* 2016<br>Chueh & Chiang 2010<br>Eghbal *et al.* 2009<br>Sharma et al. 2020	Histologic and biological study
Jalali *et al.* 2015<br>Dunlop *et al.* 2013<br>Elsharraww & Elbaghdady 2007<br>Bagheri *et al.* 2019	Unrelated to the topic
Simon *et al.* 2013<br>Tan *et al.* 2020	No symptoms of irreversible pulpitis
Orhan *et al.* 2010	Indirect pulp therapy
Asgary & Eghbal 2010	Retracted article
Yazdani *et al.* 2014	Health technology assessment
Asgary & Ehsani 2009	Case series
Zafar *et al.* 2020<br>Sadaf 2020	Systematic review/review
George 2020<br>Bakhurji 2020	Opinion article

Gemmell *et al.* 2020	Survey
Linsuwanont *et al.* 2017	Retrospective study

Table S2. Articles excluded using the EMBASE database.

Articles	Cause of exclusion
Zanini *et al.* 2017<br>Rechenberg *et al.* 2016<br>Lin *et al.* 2020	Inflammatory mediators and histological studies
Yu *et al.* 2020<br>Chompu-Inwai *et al.* 2018	Unrelated to the topic
Chen *et al.* 2020	Primary molars
Sabeti *et al.* 2021	Animal model
Wang 2020<br>Ramezani *et al.* 2020	Case Report and immature tooth
Kusumvalli *et al.* 2019	Absence of signs and symptoms of irreversible pulpitis

Table S3. Articles excluded using the Cochrane database.

ARTICLES OR MAIN ID OF CLINICAL TRIALS	CAUSES OF EXCLUSION
NCT03956199<br>NCT00748280<br>NCT04573374<br>NCT04397315<br>NCT04308863<br>NCT03186690<br>NCT04243733<br>NCT03916900<br>IRCT20151226025695N3<br>NCT03735069	Clinical trials without results reported
RCT2013030512708N1<br>NCT03168620<br>NCT04599244<br>Jalali *et al.* 2015<br>Bane *et al.* 2016<br>Kérourédan *et al.* 2017<br>Elsharrawy & Elbaghdady 2007<br>IRCT201110137790N1<br>IRCT20181021041405N1<br>IRCT2017101036699N1<br>CTRI/2019/09/021443<br>CTRI/2019/09/021443<br>RBR.5j25nm<br>ISRCTN14290358	Clinical trials/articles unrelated to the topic
Eren *et al.* 2018<br>ISRCTN14290358<br>Asgary & Eghbal 2010	Clinical outcome/pain relief
McDougal 2004	Intermediate restoration
Asgary & Eghbal 2010	Retracted

NCT04719247<br>Chen et al, 2020<br>TCTR20151017001<br>TCTR20181115015<br>ChiCTR20000032462<br>Waterhouse *et al.* 2002<br>CTRI/2019/12/022559<br>CTRI/2020/05/025148<br>PACTR201812884054327<br>IRCT138902203893N2<br>RBR-9chxvg	Primary teeth
Orhan *et al.* 2010	Absence of signs and symptoms of irreversible pulpitis
PACTR202001824413147	Access not available/pulp capping material was not a hydraulic calcium silicate cement
E Bakjuri, 2020	Abstract not available
ISRCTN84455971<br>ISRCTN84455971<br>TCTR20180612004<br>CTRI/2020/09/028105<br>CTRI/2020/03/023766<br>CTRI/2019/05/019132<br>CTRI/2020/03/023894<br>CTRI/2020/10/028640<br>CTRI/2018/06/014426	Clinical trials without published results and abstract was not available
Alawwad *et al,* 2020	Immature permanent teeth

Table S4. Articles excluded after full text reading.

Author(s)	Title	Cause of exclusion
Asgary *et al.* 2018 [38]	"Treatment Outcomes of 4 Vital Pulp Therapies in Mature Molars"	The outcome was unclear. Specific outcome for teeth with irreversible pulpitis not discernible

		from reversible pulpitis. Attempts to contact the authors were not successful.
Taha *et al.* 2017 [40]	"Assessment of Mineral Trioxide Aggregate Pulpotomy in mature permanent teeth with carious exposures"	The outcome was unclear. Specific outcome for teeth with irreversible pulpitis not discernible from reversible pulpitis. Authors were not available to provide additional clarification of the published data.
Galani *et al.* 2017 [43]	"Comparative Evaluation of Postoperative Pain and Success Rate after Pulpotomy and Root Canal Treatment in cariously Exposed Mature Permanent Molars: A Randomized Controlled Trial"	Diagnosis of pulpitis was not consistent with inclusions criteria

Table S5. Risk-of-bias in randomized controlled trials based on the Cochrane Collaboration RoB 2 tool.

	Asgary *et al.* 2013 [2]	Asgary *et al.* 2014 [3]	Asgary *et al.* 2015 [4]	Asgary & Eghbal 2013 [5]	Asgary *et al.* 2017 [6]	Kumar *et al.* 2016 [34]	Taha & Khazali 2017 [7]	Uesrichai *et al.* 2019 [8]	Koli *et al.* 2021 [41]
1.Random sequence generation	LOW	LOW	LOW	UNCERTAIN	UNCERTAIN	LOW	LOW	LOW	LOW
2.Allocation concealment	LOW	LOW	LOW	LOW	LOW	LOW	LOW	LOW	LOW
3. Blinding of participants and personnel	UNCERTAIN	UNCERTAIN	UNCERTAIN	LOW	LOW	UNCERTAIN	LOW	LOW	UNCERTAIN
4. Blinding of outcome assessment	LOW	LOW	LOW	LOW	LOW	LOW	LOW	LOW	LOW
5.Incomplete outcome data	UNCERTAIN	UNCERTAIN	UNCERTAIN	UNCERTAIN	UNCERTAIN	UNCERTAIN	LOW	LOW	LOW
6.Selective reporting	LOW	LOW	LOW	LOW	LOW	LOW	LOW	LOW	LOW
7.Other sources of bias									
7.1. Group imbalance	LOW	LOW	LOW	LOW	LOW	LOW	LOW	LOW	LOW
7.2. Sample size	LOW	LOW	LOW	LOW	LOW	LOW	UNCERTAIN	LOW	UNCERTAIN
7.3. Clinician bias	LOW	LOW	LOW	LOW	LOW	HIGH	LOW	LOW	LOW
8. Final risk of bias	FAIR	FAIR	FAIR	FAIR	FAIR	HIGH	FAIR	LOW	FAIR

Table S6. Risk of bias assessment justification for randomized clinical trials.

Author(s), year	The risk of bias justification
Asgary *et al.* 2013 [2]	**Blinding of participants and personnel**- UNCERTAIN<br>• Different protocols for each treatment<br><br>**Incomplete outcome data-** UNCERTAIN<br>• Number of teeth excluded after intraoperative assessment of pulp necrosis or hemostasis not achieved is not presented
Asgary *et al.* 2014 [3]	**Blinding of participants and personnel**- UNCERTAIN<br>• Different protocols for each treatment<br><br>**Incomplete outcome data-** UNCERTAIN<br>• Number of teeth excluded after intraoperative assessment of pulp necrosis or hemostasis not achieved is not presented
Asgary *et al.* 2015 [4]	**Blinding of participants and personnel**- UNCERTAIN<br>• Different protocols for each treatment<br>**Incomplete outcome data-** UNCERTAIN<br>• Number of teeth excluded after intraoperative assessment of pulp necrosis or hemostasis not achieved is not presented<br>• Loss to follow-up greater than 20%
Asgary & Egbhal 2013 [5]	**Random sequence generation**- UNCERTAIN<br>• Information is missing<br>**Incomplete outcome data-** UNCERTAIN<br>• Number of teeth excluded after intraoperative assessment of pulp necrosis or hemostasis not achieved is not presented the authors to clarify this point were not successful
Asgary *et al* 2017 [6]	**Random sequence generation**- UNCERTAIN<br>• Information not provided<br>**Incomplete outcome data-** UNCERTAIN<br>Number of teeth excluded after intraoperative assessment of pulp necrosis or hemostasis not achieved is not presented the authors to clarify this point were not successful
Kumar *et al.* 2016 [34]	**Blinding of participants and personnel**- UNCERTAIN<br>• Flow of the treatment protocol described was not in agreement with randomization presented in the manuscript (PRF preparation started before the beginning of pulpotomy treatment)<br>**Incomplete outcome data-** UNCERTAIN<br>• Loss to follow-up greater than 20%<br>**Clinician bias-** HIGH<br>• Non-calibration or blinding of radiographic evaluators
Taha & Khazali 2017 [7]	**Sample Size-** UNCERTAIN<br>• No statistical calculation was presented to establish the sample size.

Koli *et al* 2021 [41]	**Blinding of participants and personnel**- UNCERTAIN<br>• Tested materials and techniques are quite different and not possible to mask<br>**Sample Size-** UNCERTAIN<br>• No statistical calculation was presented to establish the sample size.

Table S7. Risk-of-bias in prospective cohort studies based on Cochrane Collaboration ROBINS-I tool.

	Qudeimat *et al.* 2017 [35]	Taha & Abdulkhader 2018 [36]	Taha & Abdelkhader 2018 [37]
1. Bias due to confounding	LOW	LOW	LOW
2. Bias in selection of participants into the study	SERIOUS*	LOW	LOW
3. Bias in classification of interventions	LOW	LOW	LOW
4. Bias due to deviations from intended interventions	LOW	LOW	LOW
5. Bias due to missing data	SERIOUS**	LOW	LOW
6. Bias in measurement of outcomes	LOW	LOW	LOW
7. Bias in selection of the reported result	LOW	LOW	LOW
Overall bias	**SERIOUS**	**LOW**	**LOW**

* Diagnosis included (i) intermittent or spontaneous, sharp or dull, localized, diffuse, or referred pain; (ii) rapid exposure to dramatic temperature changes elicited heightened and prolonged episodes of pain even after the thermal stimulus has been removed; and (iii) no clinical symptoms but pulpal bleeding produced by caries excavation.

** A specific end-point of the follow-up is not presented.

Table S8. Risk-of-bias in prospective cohort studies based on the Newcastle-Ottawa Scale.

		Qudeimat *et al.* 2017 [35]	Taha & Abdulkhader 2018 [36]	Taha & Abdelkhader 2018 [37]
**Selection**	1. Representativeness of the exposed cohort	_ a	_ a	★
	2. Selection of the non-exposed cohort	_ b	_ b	_ b
	3. Ascertainment of exposure	★	★	★
	4. Demonstration that outcome of interest was not present at start of the study	★	★	★
**Comparability**		_ b	_ b	_ b
**Outcome**	1. Assessment of outcome	★	★	★
	2. Was follow-up long enough for outcomes to occur	_ c	★	★
	3. Adequacy of follow up of cohorts	★	★	★
Overall merit		4	5	6

a. Age range includes only young patients (age range 10 to 17 years old).
b. Non-exposed group inexistent.
c. A specific follow-up time length was not presented at the beginning of the study. The patients were evaluated only once, at different time lengths.
